# Nutrition in Gynecological Diseases: Current Perspectives

**DOI:** 10.3390/nu13041178

**Published:** 2021-04-02

**Authors:** Michał Ciebiera, Sahar Esfandyari, Hiba Siblini, Lillian Prince, Hoda Elkafas, Cezary Wojtyła, Ayman Al-Hendy, Mohamed Ali

**Affiliations:** 1 Second Department of Obstetrics and Gynecology, Center of Postgraduate Medical Education, 01-809 Warsaw, Poland; michal.ciebiera@cmkp.edu.pl; 2 Department of Surgery, University of Illinois at Chicago, Chicago, IL 60612, USA; sesfan2@uic.edu (S.E.); helkaf2@uic.edu (H.E.); 3 Department of Obstetrics and Gynecology, University of Chicago, Chicago, IL 60637, USA; hsiblini@bsd.uchicago.edu (H.S.); aalhendy@bsd.uchicago.edu (A.A.-H.); 4 Biological Sciences Division, Public Health Sciences, University of Chicago, Chicago, IL 60637, USA; lprince@uchicago.edu; 5 Department of Pharmacology and Toxicology, Egyptian Drug Authority (EDA), Cairo 15301, Egypt; 6 International Prevention Research Institute-Collaborating Centre, Calisia University, 62-800 Kalisz, Poland; cezary.wojtyla@gmail.com; 7 Clinical Pharmacy Department, Faculty of Pharmacy, Ain Shams University, Cairo 11566, Egypt

**Keywords:** nutrition, gynecological diseases, infertility, PCOS, uterine fibroids, endometriosis, microbiome, infection, cervical cancer, endometrial cancer, ovarian cancer, dysmenorrhea, diet, nutrients, complementary and alternative medicine

## Abstract

Diet and nutrition are fundamental in maintaining the general health of populations, including women’s health. Health status can be affected by nutrient deficiency and vice versa. Gene–nutrient interactions are important contributors to health management and disease prevention. Nutrition can alter gene expression, as well as the susceptibility to diseases, including cancer, through several mechanisms. Gynecological diseases in general are diseases involving the female reproductive system and include benign and malignant tumors, infections, and endocrine diseases. Benign diseases such as uterine fibroids and endometriosis are common, with a negative impact on women’s quality of life, while malignant tumors are among the most common cause of death in the recent years. In this comprehensive review article, a bibliographic search was performed for retrieving information about nutrients and how their deficiencies can be associated with gynecological diseases, namely polycystic ovary syndrome, infertility, uterine fibroids, endometriosis, dysmenorrhea, and infections, as well as cervical, endometrial, and ovarian cancers. Moreover, we discussed the potential beneficial impact of promising natural compounds and dietary supplements on alleviating these significant diseases.

## 1. Introduction

Gynecological diseases are diseases of the female reproductive organs; these diseases are considered a public health and social problem. These diseases include benign and malignant tumors, infections, and endocrine disorders. All these diseases significantly impact women’s quality of life, and many of them, unfortunately, are still lacking efficient treatment plans. Promoting both primary and secondary prevention is essential for the sake of these afflicted women and their reproductive health [1]. Sometimes, applying such preventive approaches is as or even more important than curative procedures. Educating patients about the significance of a healthy lifestyle and explaining hygienic and dietary measures are among these imperative procedures.

Gene–nutrient interactions are central contributors to health management and disease prevention. Nutrigenomics and nutrigenetics are defined as sciences that investigate the relationship between genetic variations and nutrient requirements [2]. Interestingly, it was recently reported that nutrients can drive epigenetic changes that can influence such requirements. Nutrition can alter gene expression, as well as the susceptibility to several diseases, including cancer, through genetic and epigenetic changes [3]. During the past decade, it has become clearer that nutrition can exert imprinting effects on the human genome, with many studies indicating that early life nutrition could influence the risk of developing chronic diseases in adulthood [4,5]. For example, with regard to the role of nutrition in cancer development, existing evidence suggests that dietary components can impact disease pathogenesis via the activation of tumor suppressor genes, cellular apoptosis, protein translation, and noncoding microRNAs (miRNAs) with roles in messenger RNA (mRNA) stability and translation [6,7]. In this article, we summarized published research in the public domain regarding the existing correlation between nutrients and dietary supplements with common gynecological diseases, highlighting the essential role of nutrients and dietary supplements in halting disease progression.

## 2. Infertility

Infertility is estimated to affect 8–16% of reproductive-age couples worldwide [8]. Lifestyle and nutritional factors have been shown to be important elements of normal reproductive function [9,10]. The literature exploring the relationship between diet and infertility has expanded over the past decade. Studies agree that the intake of folic acid is recommended for the prevention of neural tube defects and has been shown to be related to a lower frequency of infertility and a lower risk of pregnancy loss [11,12]. Further nutritional components or types of diet have been studied in relation to female infertility, including the Mediterranean diet, fats, vitamins, caffeine, smoking, alcohol, and, more recently, probiotics [11].

### 2.1. Mediterranean Diet

The Mediterranean diet is a diet rich in vegetables, fruits, whole grains, legumes, nuts, and olive oil and low in red meat. It has proven to be beneficial in several aspects of human health in general [13] and has also been studied in relation to fertility [14]. Previously, Vujkovic et al. studied the association between a preconception diet and in vitro fertilization (IVF) in a cohort of subfertile couples in the Netherlands and showed that adherence to the Mediterranean diet is associated with a higher chance of pregnancy [15]. A similar effect was later confirmed in a Dutch cohort of couples undergoing first-time in vitro fertilization (IVF), and the authors explained that the high fat content of vegetable oil as part of this diet could be the driving force behind this association. Subsequently, a prospective cohort study of 244 non-obese women who underwent their first IVF in Athens showed that adherence to the Mediterranean diet is associated with an increased chance of clinical pregnancy and live birth [16]. Results from the Nurses’ Health Study cohort, which included 438 reported infertilities related to ovulation disorders, showed a significant association between female fertility and consumption of low-glycemic carbohydrates, monounsaturated fatty acids, proteins of plant origin, and supplements with iron, folate, and vitamins [17]. The authors concluded that adherence to such components, which are essentially present in the Mediterranean diet, is associated with a lower risk of ovulatory infertility [17].

### 2.2. Fats

Long-chain omega-3 fatty acids seem to improve female infertility, although it is unclear whether environmental toxins in such food sources, such as fish, can reduce this benefit [18]. In a prospective study of a cohort of 1228 women attempting pregnancy followed for up to six menstrual cycles, the preconception plasma phospholipid fatty acid level was measured at baseline [18]. The authors concluded that monounsaturated fatty acids (MUFAs) are associated with increased fecundability or a shorter time to pregnancy, whereas polyunsaturated fatty acids show the opposite effect. The role of polyunsaturated fatty acids (PUFAs) to decrease fecundability may be due to their effect on androgen synthesis, and androgens have been associated with ovulatory disorders such as polycystic ovary syndrome. Fatty acids are also thought to effect fecundability through changes in insulin sensitivity and inflammation, as these pathways also influence ovulatory function [19].

### 2.3. Vitamins

Despite promising evidence from preclinical animal model studies, vitamin D deficiency does not appear to influence human fertility [20,21]. Among women attempting pregnancy in the Nurses’ Health Study II (NHSII) cohort, higher intake of vitamin D was not associated with a risk of ovulatory infertility [22]. Similarly, among another large cohorts of women with no history of infertility but with one to two prior pregnancy losses, no association was found between baseline vitamin D levels or vitamin D deficiency and fecundability [23].

A more recent topic of interest is the role of antioxidant consumption based on evidence from experimental association between low antioxidant status and infertility [24]. In one study, increased intake of beta carotene, vitamin C, and vitamin E was associated with a shorter time to pregnancy (TTP), but the effects varied with the body mass index (BMI) and age. A shorter TTP was observed among women with a body mass index (BMI) of <25 kg/m^2^ with increasing vitamin C, among women with a BMI of ≥25 kg/m^2^ with increasing beta carotene, among women aged <35 years with increasing beta carotene and vitamin C, and among women aged ≥35 years with increasing vitamin E [25].

### 2.4. Probiotics

Considerable attention is lately being given toward probiotics and the effect of the gut microbiome on diseases [26]. Nevertheless, the role of the microbiome in infertility and the role of probiotics in infertility management have not been extensively studied. Lactobacilli are the most studied probiotic bacteria, and they show several mechanisms in protecting the vaginal environment, including production of lactic acid that deters pathogens by lowering the pH and yielding an acidic environment to the cervico-vaginal mucus [27]; production of bacteriocins, which are antimicrobial peptides and proteins that protect against microbial invasion; and enhancement of immunomodulation by producing H_2_O_2_ and stimulating anti-inflammatory action [28]. In a recent review article, Younis et al. drew attention to the importance of further exploring in future clinical research the role of probiotics in managing infertility [29]. Bhandari et al. demonstrated that Lactobacillis plantarum works to competitively exclude sperm-agglutinating Escherichia coli (*E. coli*) bacteria. They treated a mice model with *E. coli* for 10 days intravaginally and then administered *L. plantarum* to find out that the fertility of this group was comparable to that of the control group, which reinforces the hypothesis that Lactobacillus probiotics may be used as an infertility therapeutic agent [30]. However, further research at the clinical level is needed to confirm these findings.

## 3. Polycystic Ovary Syndrome

Polycystic ovary syndrome (PCOS) is a complex and common hormonal condition in women of reproductive age, characterized by ovulatory dysfunction, chronic anovulation, altered menstruation, and ovarian small cysts on one or both ovaries, which may affect fertility [31]. It is found in approximately 5% to 10% of women aged between 18 and 44 years, making it among the most widespread diseases of reproductive-age women. Based on the previous literature, PCOS is associated with other common disorders, such as insulin resistance, obesity, type 2 diabetes, hypertension, endometrial cancer, and hyperandrogenemia. Indeed, most women with PCOS have insulin resistance [32,33,34].

Although the actual cause of PCOS remains unclear, evidence points to the role of environmental factors, including lifestyle and dietary habits, in the prevention and treatment of PCOS. Therefore, considering these factors may propose new therapeutic strategies for PCOS patients [35,36]. One of the most prominent approaches in treating PCOS is diet therapy for the sake of reducing insulin resistance and reproductive dysfunction. Considering the association of PCOS with obesity and insulin resistance, it should be noted that approximately a 5% to 10% decrease in weight may increase reproductive activity. This might be achieved by weight loss, a decrease in the intake of foods with a high glycemic index and foods rich in fatty acids, and intake of sufficient omega-3, vitamin D, and chromium [35]. There are several studies considering the effects of these nutritional components on the control of PCOS, which we will discuss in this review.

First, foods rich in fat, mainly saturated fatty acids, increase the risk of insulin resistance and its related complications, while diets rich in unsaturated fatty acids decrease the risk of these diseases [35,37]. In this manner, the intake of omega-3 unsaturated fatty acids reduces the risk of PCOS in women with insulin resistance [35]. Moreover, another study indicated that the intake of unsaturated fatty acids affects the levels of pregnanediol 3-glucuronide in cases with PCOS, although the levels of sex hormones do not alter [38].

Current evidence has revealed the role of vitamin D in different metabolic pathways, including the insulin signaling pathway [39]. Previous studies have shown that the vitamin D signaling pathway directly contributes to the activation of the insulin receptor gene [39]. Hence, sub-optimal vitamin D levels may be associated with the pathogenesis of insulin resistance and PCOS [39,40]. In this context, a recent systematic review and meta-analysis demonstrated the association between vitamin D levels and the metabolic profile, including high-density lipoprotein (HDL-C), fasting blood glucose, and insulin, in PCOS women. Furthermore, vitamin D levels had a positive relationship with sexual hormone binding globulin (SHBG). These findings suggested an important role of vitamin D supplementation in infertile women with PCOS who undergo ovarian stimulation [41].

Recently, attention has shifted toward the effect of vitamin D on ovarian function in PCOS. However, the underlying mechanism of vitamin D on ovarian function is still not fully determined. One possible mechanism is the role of vitamin D in alleviating the inflammatory pathways causing insulin resistance [35]. Previous studies have also indicated the existence of a vitamin D receptor in the granulosa cells of ovaries [42,43]. Moreover, another study showed that the anti-Müllerian hormone (AMH) promoter is under vitamin D down-regulation. AMH is produced by growing follicles, and its excess excretion is linked to PCOS. Therefore, it seems likely that vitamin D supplementation can affect ovarian function and alleviate PCOS [44]. A clinical trial reported that vitamin D supplementation in PCOS women with vitamin D deficiency was related to lower levels of AMH [45]. Consequently, it is tempting to speculate that vitamin D supplementation may be effective in PCOS patients.

Many natural anti-androgen foods have driven scientists’ attention to their effects on PCOS therapy. Considering the effect of high insulin levels on the production of testosterone and the high levels of this androgen in PCOS women, improving insulin sensitivity by changing the diet and lifestyle may be regarded as the first-line treatment in this disorder [46,47]. According to previous studies, a low-carbohydrate diet is correlated with a lower risk of metabolic diseases, including insulin resistance, type 2 diabetes, and obesity, along with a lower risk of reproductive disorders [48,49,50]. However, other studies have shown that a low-carbohydrate diet does not affect the metabolic profile and levels of sex hormones in PCOS women [36,51].

Here, we mention some of the most notable natural anti-androgen foods used in PCOS studies. Soybean comprises isoflavone and phytoestrogens, which are critical in modulating many androgens in the human body [52]. A study showed that soybean phytoestrogen decreased the level of testosterone after three months [53]. Green tea with a high amount of antioxidants was used in a study on PCOS women and promoted insulin sensitivity and lowered the levels of testosterone [54]. Licorice is another phytoestrogen that alleviates the symptoms of PCOS patients and reduces the levels of testosterone [55]. Collectively, natural anti-androgen foods can be considered in the lifestyle to lower testosterone levels and alleviate PCOS.

Flavonoids are polyphenolic compounds of plants that have antioxidant, antiestrogenic, and antidiabetic properties [56]. These natural compounds are emerging as important mediators in the pathogenesis of many reproductive disorders, such as PCOS. Among them, quercetin as a bioflavonoid with antioxidant activity is effective in PCOS therapy [57]. It is reported that quercetin may reduce many androgens in rats [58]. Quercetin also reduced insulin resistance in an animal model of PCOS [59].

A clinical trial showed that quercetin improves insulin resistance and hormonal profile in PCOS women [60]. Another study demonstrated that a daily dose of 1000 mg of quercetin for 12 weeks was effective in alleviating PCOS features [61]. Moreover, previous studies have also indicated the potential effects of other flavonoids, such as resveratrol and soy isoflavones, in the treatment of PCOS through the regulation of steroidogenesis, metabolic parameters, ovarian cysts, and follicular development [62,63,64].

Several studies have suggested the role of minerals in the pathogenesis of PCOS [65]. In this regard, chromium is one of the most important minerals playing a role in carbohydrate and lipid metabolism, whose deficiency is observed in patients with type 2 diabetes. Subsequently, it seems plausible that chromium deficiency increases the risk of insulin resistance [66]. Furthermore, it is evident that PCOS patients have a lower level of chromium, which is associated with insulin resistance [67]. Interestingly, chromium supplementation with a dose of 200 µg for three months improved glucose tolerance in PCOS patients. However, it did not change the reproductive function [68]. Another study demonstrated that chromium supplementation with a daily dose of 200 µg for eight weeks improved insulin resistance and other metabolic parameters in PCOS women compared to the placebo [69]. It is also reported that chromium picolinate might decrease hirsutism and improve the symptoms of PCOS [70].

Other minerals that participate in reproductive function and are important in the pathogenesis of PCOS are calcium, selenium, zinc, and magnesium [65]. Calcium is a key mineral involved in follicular development and oocyte maturation [71]. It is demonstrated that calcium plays an important role in the insulin signaling pathway. Thus, calcium deficiency may be correlated with insulin resistance and the following PCOS. Obese women with PCOS have lower levels of calcium compared with healthy individuals. Interestingly, vitamin D receptor (VDR) is associated with calcium homeostasis [66,71,72]. A study reported that supplementation of calcium in combination with vitamin D and metformin 1500 mg for six months decreases the body mass index of PCOS patients. The authors also observed that this supplementation improves follicular development and the fertility rate, although the results were not statistically significant [73].

Selenium is an antioxidant mineral important for the development and activity of reproductive tissues [74]. Lower levels of selenium as well as a higher amount of free radicals have been reported in PCOS patients, which cause a higher production of androgens, luteinizing hormone (LH), and testosterone [75]. Interestingly, selenium also has insulin-like activities [76]. Therefore, it may affect carbohydrate and lipid metabolism. A clinical trial showed that selenium supplementation at a daily dose of 200 µg for eight weeks alleviates insulin resistance in PCOS patients [77]. Hence, selenium supplementation seems to have potential in the adjustment and improvement of insulin resistance and PCOS.

Another mineral, zinc, is a cofactor for many enzymes in carbohydrate and lipid metabolism [78]. Therefore, it also plays a key role in insulin resistance and PCOS. It is reported that women with PCOS represent lower levels of zinc [79,80]. Collectively, zinc supplementation may provide an adjunctive nutritional treatment for inducing insulin sensitivity in women with PCOS. Magnesium as a regulator of ATP use is also an important trace element for the metabolism of insulin [81]. A low level of magnesium was observed in women with insulin resistance and high levels of testosterone [82]. It should be noted that only limited studies have investigated the association between magnesium levels and the pathogenesis of PCOS. Hence, their relationship remains unrecognized [65].

## 4. Uterine Fibroids

Uterine fibroids (UFs) are the most common gynecological tumors and a major cause of gynecological morbidity in reproductive-age women [83,84]. They are also the leading cause of hysterectomies in the United States, with more than 200,000 hysterectomies yearly [85]. The annual costs attributed to UFs range between $5.9 and $34.4 billion per year in the United States alone and hundreds of billions worldwide [86]. Although UFs are benign tumors, they can cause a myriad of symptoms and outcomes, including pelvic pain, abnormal uterine bleeding, bladder dysfunction, and even infertility [87]. Despite the high morbidity and cost associated with UFs, the exact pathophysiology is not completely delineated [88], yet there are theories and reports of associated risk factors. Some of these risk factors include an increased body mass index, early age at menarche, nulliparity, vitamin D deficiency, and African American ethnicity [89]. A growing body of research has shed light on increasing evidence that dietary factors may play a role in UF etiology and growth [90]. This is hypothesized to be due to their ability to modify endogenous hormones as well as their inflammatory or anti-inflammatory effects.

### 4.1. Fats

Fats have been extensively studied in relation to UFs, seeing their effect on the inflammatory milieu. For example, trans fats are reported to influence levels of interleukin 6 (IL-6) and other inflammatory markers [91]. Fats also have an effect on hormone levels, as a meta-analysis of 13 intervention studies reported that reducing fat consumption results in lower serum estradiol levels [92].

As previously mentioned, the African American race is considered a risk factor for UFs, and the time of onset is estimated to be 10 to 15 years earlier for this race cohort [93]. In addition, the source of dietary fat intake is shown to be generally different between black and white women in the United States, with black women consuming more fat from meat and fish and less from dairy products as compared to white women [94]. The Black Women’s Healthy Study (BWHS) [95] was the first prospective study comprising solely a black women cohort to study the association between dietary fat and UF risk. More than 12,000 African American women were followed for eight years, with 2695 having fibroids that were self-reported, ultrasound detected, or detected during hysterectomy or other surgery. Wise et al. studied data from the BWHS and reported an increased risk of fibroids with the intake of specific ω-3 polyunsaturated fatty acids (PUFAs) but no consistent association with total fat or other fat subtypes, with the exception of total monounsaturated fatty acid (MUFA) intake, for which a positive association was identified [96]. Nevertheless, fish consumption was the main source of PUFAs in this population, so results may have been confounded by environmental contaminants in fish intake. Biomarker measurements of exposure to pollutants in future studies could help differentiate the extent to which the association is explained by pollutants or fatty acids themselves.

Moreover, the BWHS did not measure circulating fatty acids (FAs) that reflect dietary intake and FA metabolism and reflect the internal dose more precisely than estimations based on diet assessment questionnaires, and UF case identification has been based on self-reporting. To expand on the literature, Wise et al. studied this association in the Study of Environment, Lifestyle, and Fibroids (SELF), in which a prospective cohort of African American women underwent serial ultrasound screening for UF incidence during a five-year period [97]. Findings were consistent with those from the BWHS, in which higher intake of marine ω-3 PUFAs are associated with a 13–21% increased risk of UFs.

A more recent prospective study done by Harris et al. examined a cohort aged 25–42 in the NHS II and studied the erythrocyte membrane FA levels of a subset of women [98]. This allowed considering dietary intake and endogenous synthesis and transformation of FAs instead of serum FA levels alone, since erythrocytes reflect long-term intake better than plasma. This study showed an inverse association between total ω-3 PUFAs and a positive association of trans FAs and the onset of uterine fibroids. Moreover, it has been shown that there is an estrogenic or inflammatory effect of dietary fat reflected by the reduction in women’s quality of life [92] and by an increase in T helper cytokines associated with fat intake, which is hypothesized to promote chronic inflammation and fibroid tissue growth [99]. However, total trans FAs were associated with higher odds of fibroids. In contrast, an Italian case–control study of 843 histologically confirmed UF cases and 1557 controls reported no association between butter, margarine, or oil intake during the year prior to the study and fibroid risk [100]. Also consistent with these null results was a cross-sectional study of Japanese women including 54 UF cases and 234 controls that reported no association between all fat subtypes and fibroid risk [101]. Islam et al. stated that the myometrium has a higher amount of arachidonic acid than UFs, with alpha-linolenic acid (ALA) being higher in UFs. Treatment with eicosapentaenoic acid (EPA) and docosahexaenoic acid (DHA) reduced the monounsaturated fatty acid content in UFs and controls. However, these did not reflect changes in the mRNA expression of extracellular matrix (ECM) components. Omega-3 fatty acids reduced the levels of sterol regulatory molecules (e.g., ATP-binding cassette sub-family G member 1 (ABCG1) or ATP-binding cassette transporter member 1 (ABCA1)) in both cell types. It also reduced a cytochrome 450 family member CYP11A1, the mitochondrial enzyme that catalyzes the conversion of cholesterol to pregnenolone. The authors concluded that omega-3 fatty acids modulate the lipid profile, mechanical signaling, and cellular lipid accumulation in UFs [102].

### 4.2. Vegetables, Meat, and Phytochemicals

National surveys have shown that African Americans have a lower intake of vegetables and fruits [103,104]. Several studies have demonstrated that diets rich in vegetables, fruits, and dairy foods play a positive and sometimes protective role in UFs [105] and, conversely, that substantial intake of red meat might increase the risk of fibroids. Fruits and vegetables are good sources of vitamins, antioxidants, and phytochemicals, and numerous studies have shown that they may decease fibroid risk. Wise et al. evaluated the association between fruit and vegetable intake and UF risk in the BWHS [105]. They studied specific components such as carotenoids, folate, fiber, and vitamins A, C, and E. Results suggested that fruit intake is inversely associated with UF risk, with the highest reduction observed for citrus fruit intake. It has been hypothesized that citrus fruit may reduce UF risk through pathways mediated by sex hormones or by inhibition of sex hormones receptors.

In a case–control study in China, He and co-authors confirmed the reduced UF risk with fruits and vegetables but found no association with meat intake [106]. The authors hypothesized that the protective role of a high intake of vegetables and fruits may be related to fibers and lycopene. Fibers can influence sex hormone and bile acid metabolism by interrupting the enterohepatic circulation. Lycopene, which makes up the red pigment in tomatoes, has been proven to decrease fibroid size in a Japanese quail study, but these results are yet to be proven in humans [107]. Phytoestrogens, which are bioactive nutrients found in plants such as soy, have been found to have moderate estrogen and antiestrogen activity [108]. Because of the presence of the phenol ring, soy isoflavone, a type of phytoestrogen, can bind to the estrogen receptor and compete with estradiol. Recently, an in vitro study explored the effects of quercetin and indole-3-carbinol (I3C) on ECM expression, cell migration, and proliferation in human myometrial and UF cells [109]. Quercetin is a flavonoid with known antifibrotic effects found in most edible fruits and vegetables, such as tea, lemon, tomato, onion leaves, and strawberry, while I3C is a naturally occurring glucosinolate in cruciferous vegetables. Results showed that both treatments significantly reduce the expression of ECM markers collagen type I and fibronectin but not versican. Moreover, the treatments inhibited UF cell migration [109].

Anthocyanins are water-soluble flavonoid pigments that are abundant in blueberries, raspberries, and strawberries [110]. Strawberries have anti-inflammatory, anti-oxidative, anti-proliferative, and genomic-protective effects [111,112]. Islam et al. explored the effect of different strawberry Alba cultivar extracts on apoptosis, fibrosis, and oxidation in the myometrium and UF cells. Results showed that anthocyanin-rich strawberries induce apoptosis but suppress glycolysis and fibrosis in UF cells. Following strawberry treatment, the authors observed an increase in reactive oxygen species levels in UFs. Additionally, the anthocyanin-rich extract significantly reduced fibronectin, collagen, and versican mRNA expression in UF cells [113]. In addition, a recent study tested five different strawberry cultivars to identify the one with the best anti-UF effects. The authors found that Alba and Romina cultivars presented the best results: they decreased collagen 1A1, fibronectin, versican, and activin A mRNA expression in UF cells [112]. In vitro studies have shown that curcumin, which is abundantly found in turmeric, prevents fibroid growth as it inhibits UF cell proliferation by regulating the apoptotic pathway [87]. Isoliquiritigenin, which is abundantly found in liquorice, and soybean have been reported to induce growth inhibition and apoptosis of UF cells [114]. On the other hand, studies have also shown both stimulatory and inhibitory effects of genistein, which is abundantly found in soybeans and fava beans, on fibroid cell proliferation [115,116]. Lower concentrations (≤1 μg/mL) stimulated proliferation, whereas higher concentrations (≥10 μg/mL) significantly inhibited proliferation, decreased proliferating cell nuclear antigen (PCNA), and increased apoptosis of both myometrial and leiomyoma cells. Finally, resveratrol, found in mulberries, peanuts, and grapes, is shown to be inversely associated with the proliferation of fibroids also via inducing apoptosis of UF cells in vitro [87].

Al Hendy et al. showed that intake of epigallocatechin gallate (EGCG), a green tea extract, reduces fibroid size [87]. Thirty-nine reproductive-age women with symptomatic UFs were studied, and although the placebo group was found to have an increase in fibroid size, those randomized to the green tea extract treatment showed an average of 32.6% (*p* = 0.0001) reduction in UF volume. This was attributed to EGCG’s inhibitory effect on the proliferation of leiomyoma cells and induction of apoptosis, as was proven by Al Hendy et al. preclinically.

### 4.3. Dairy Foods and Vitamins

National surveys have shown that African Americans consume less dairy food than white Americans do, and they are less likely to take vitamin supplements as well [117]. Dairy foods have antitumorigenic constituents, including calcium, vitamin D, butyric acid, and milk proteins [118]. Yet, milk may contain estrogen and progesterone that may increase the risk of hormone-dependent tumors [119]. In the BWHS cohort, Wise et al. prospectively studied the effect of dairy food on UF risk and found that women who had four of more dairy servings per day had a 30% reduction in the incidence of fibroids [120]. Calcium, phosphorus, and the calcium-to-phosphorus ratio (as an indicator of calcium bioavailability) were inversely associated with UF risk. It is hypothesized that calcium may reduce fat-induced cell proliferation. In this study, Wise et al. did not find an effect of dietary vitamin D, and this was attributed to the fact that the largest bioavailable sources of vitamin D are derived from sun exposure and supplements. In a later study, Wise identified single-nucleotide polymorphisms in genes involved in vitamin D metabolism that were significantly associated with UFs [121]. It has also been shown African American women have lower serum vitamin D levels than white Americans, which could explain the increased risk of UFs [122]. Al Hendy et al. observed that vitamin D could be a potent antiestrogenic agent that reduces the expression of sex steroid receptors and consequently the risk of UFs [123]. Vitamin D has been shown to be a potent antitumor agent inhibiting UF cell proliferation and decreasing UF size in in vivo animal models as well as several clinical trials [122,124,125]. Mechanistically, vitamin D might exert its anti-UF effects via induction of DNA repair genes and amelioration of DNA damage both in UF cells and in myometrial cells at risk of developing UFs [126,127,128]. More recently, Sheng et al. published a protocol for the first open-label randomized controlled trial to evaluate whether supplementation with vitamin D can reduce the risk of UFs in reproductive-age women, and future results of this study could provide new evidence of the benefit of vitamin D intake [129].

### 4.4. Pollutants and Metaloestrogens

As explained previously, fibroid development is mostly mediated by estrogen and progesterone receptors. Many pollutants resemble these steroid hormones and affect these receptors as endocrine-disrupting chemicals (EDCs). As described in a recent review, these include phthalates, parabens, environmental phenols, alternate plasticizers, diethylstilbestrol, organophosphate esters, and tributyltin. The US National Institute of Environmental Health Sciences (NIEHS) defines EDCs as “chemicals that interfere with the body’s endocrine system and produce adverse developmental, reproductive, neurological and immune effects.” EDCs have been described to bind to nuclear receptors, such as estrogen receptors, and alter hormone functions by mimicking endogenous hormones and/or blocking them from interacting with their receptors. EDCs may also induce genomic and nongenomic signaling. For example, bisphenol A and diethylstilbesterol have been shown to activate nongenomic signaling through estrogen receptors [130]. Epidemiologic studies have shown that exposure to certain EDCs is associated with increased fibroid risk and severity [131].

Some heavy metals, which are mostly present in tobacco smoke, polluted air, seafood, and leafy green vegetables, are also associated with increased fibroid risk. The Endometriosis: Natural History, Diagnosis, and Outcomes (ENDO) study demonstrated a direct link between fibroids and increased serum levels of cadmium and lead and urinary cobalt levels [132]. Heavy metals as metaloestrogens activate the estrogen receptor in the absence of estradiol and affect the hypothalamic–pituitary–ovarian axis as do endocrine-disrupting compounds [133].

## 5. Endometriosis

Endometriosis is an inflammatory and estrogen-dependent gynecological disorder characterized by the proliferation of endometrial cells outside the uterine cavity [134]. Indeed, endometrial cells migrate from their original site, the uterus, to other organs and produce endometrial-like tissues in various anatomical sites outside the uterine cavity, particularly the ovaries and the peritoneum [134,135]. Although the symptoms of endometriosis are not specific and most of them are similar to symptoms of other gynecological diseases, it may cause pelvic pain and infertility. Exosomes act as biomarkers for female reproductive disease diagnosis and therapy [136]. Moreover, it should be noted that endometriosis creates a significant burden in terms of health expenditure and quality of life all over the world [136]. It is a disorder with approximately 3 to 11 years of diagnostic delay, resulting in the dysfunction of the reproductive cycle in reproductive-age women. The exact prevalence of endometriosis is not determined due to a lack of proper non-invasive diagnostic techniques, but it is estimated that about 10% of women of reproductive age suffer from endometriosis. Furthermore, its prevalence rises to approximately 20% to 50% in women with pelvic pain or infertility [136,137,138].

Endometriosis is a multifactorial disorder that involves genetic and immunologic pathways, contraction of the smooth muscle, and inflammation, as well as environmental factors, including dietary habits and nutrition components. According to previous studies, the development of endometriosis requires alterations in several biological pathways for disease establishment [139,140]. The present work aimed to summarize the biological effects of nutrition components, including omega-3, omega-6, vitamin D, N-acetylcysteine, flavonoids, and L-carnitine, on the prevention and treatment of endometriosis.

Foods rich in omega-6 fatty acids, such as red meat, are correlated with higher levels of estradiol and estrone sulfate, which is linked to higher concentrations of steroids, inflammation, and the development of endometriosis [141]. Instead, supplementation with omega-3 may decrease the growth of endometrial implants and the production of inflammatory factors, particularly in patients with stage III or IV endometriosis [142].

Vitamin D is a classic regulator of inflammatory pathways and has been widely studied in the field of endometriosis. Macrophages, lymphocytes, and dendritic cells (DCs) express enzymes that use this vitamin [143]. It has been shown that these cells express CYP27B1, while DCs also express CYP2R1, which are both key enzymes in vitamin D metabolism. All these cell types can convert hydroxy vitamin D3 (25(OH)D3) into bioactive dihydroxy vitamin D3 (1,25(OH)2D3), enabling them to respond not only to the active vitamin D metabolite but also to its precursors]. Vitamin D boosts the shift away from Th1-type responses to a Th2-type immunity by repressing the secretion of IL-12, IL-2, tumor necrosis factor (TNF), and γ-interferon by macrophages, T cells, and DCs [143]. Therefore, the active form of vitamin D may act in the endometriosis lesion by lowering the production of prostaglandins and inflammatory cytokines [143]. A clinical trial demonstrated that patients with dysmenorrhea treated with a dose of 300,000 IU of vitamin D had lower pain along with lower use of nonsteroidal anti-inflammatory drugs (NSAIDs) [144]. However, another clinical trial reported that vitamin D supplementation at a dose of 50,000 IU weekly for 12 weeks did not affect endometriosis-related pain [145]. Hence, further studies are required in this area of research.

N-acetylcysteine, also known as acetylcysteine, can effectively reduce inflammation and alleviate endometriosis. Interestingly, foods with N-acetylcysteine, including onions, garlic, wheat germ, broccoli, and Brussels sprouts, are reported to have the ability to control cell proliferation and oxidative stress in endometriotic cells [146]. A study observed that the size of the endometrioma in patients supplemented with N-acetylcysteine at a dose of 1800 mg reduced significantly [147].

Studies have shown that quercetin acts as a natural flavonoid in endometriosis therapy [148]. A study reported that quercetin affected the hypothalamic–pituitary–gonadal (HPGA) axis in an animal model of endometriosis. Therefore, quercetin decreased the levels of Luteinizing hormone (LH) and follicle-stimulating hormone (FSH). Furthermore, it reduced the levels of estrogen and progesterone receptors [149]. Resveratrol is a polyphenol ingredient found in grapes, peanuts, and cocoa with anti-inflammatory and antioxidant activities. A study reported that resveratrol supplementation reduced the size of endometriomas in an animal model. Moreover, it reduced the levels of vascular endothelial growth factor (VEGF) in endometrial tissue, which is efficient for endometriosis therapy [150]. A randomized exploratory trial in infertile patients with endometriosis (stage III–IV) within the window of implantation revealed that receiving resveratrol (400 mg) for 12–14 weeks significantly attenuated the levels of VEGF and TNF-α genes and protein in the ectopic endometrium compared with the placebo group [151]. Several studies have reported that sulforaphane (SFN), an isothiocyanate in cruciferous vegetables such as cauliflower, cabbage, and broccoli, has antioxidative, antitumor, anti-inflammatory, and immune-enhancing effects [152,153]. Zhou et al. reported that administrating SFN in an endometriosis rat model for three weeks dose-dependently attenuated the volumes of the adhesion score and endometriotic foci. Further, post-treatment of SFN repressed levels of VEGF, interferon gamma (IFN-γ), TNF-α, IL-6 and IL-10 in plasma and peritoneal fluid and regulated the expression of cleaved caspase-3, bcl-2, Bax, and VEGF in endometrial tissue by repression of the PI3K/Akt pathway [152]. These studies suggest that flavonoids may inhibit ectopic endometrium growth.

L-carnitine is an amino acid analogue involved in fatty acid oxidation and energy metabolism [154]. Studies have shown that L-carnitine supplementation acts as a double-edged sword in the progression of endometriosis. For instance, it was reported that L-carnitine intensified an already presented endometriotic lesion when cells expressed estrogen receptors, while it improved this situation when cells did not express estrogen receptors. Clearly, the underlying mechanism is linked to the cellular features of cells arising from the endometrium [155].

Altogether, there are many studies on the role of different nutrients in endometriosis, which provide promising approaches to disease control. It seems that foods rich in omega-3, N-acetylcysteine, and polyphenol, in addition to decreased consumption of omega-6 fatty acids, may lower the plausible risk of endometriosis. Therefore, dietary education appears to be a promising strategy for the control of the disease.

## 6. Vaginal Microbiome, Nutrients, and Female Reproductive Tract Infections

The worldwide burden of reproductive tract infections (RTIs) is a vast and major public health concern, particularly in developing countries where RTIs are widespread [156]. RTIs, except for human immunodeficiency virus (HIV), are considered the next major cause of disease burden (after maternity-related causes) in young women in developing countries. RTIs involve three sets of infections [156,157]: sexually transmitted infections (STIs), infections that arise from the overgrowth of organisms usually present in the reproductive tract, and, finally, infections connected with therapeutic plans, including abortion and insertion of intrauterine devices.

Female RTIs usually start in the lower genital tract as vaginitis or cervicitis and may exhibit irregular vaginal discharge, genital discomfort, itching, and burning sensation with urination. RTIs causes a heavy burden on women if untreated, and they can cause serious infertility, cervical cancer, ectopic pregnancy, menstrual disturbances, pregnancy wastage, and low-birth-weight babies [158].

An environment’s microbiota consists of resident bacteria, viruses, fungi, protists, and archaea. Either culture-based or sequence-based techniques can distinguish the bacterial microbiome. Both methods have been used to define various sites within the women’s reproductive tract, including the vagina, cervix, and uterus. While sequence-based techniques are not routinely used to recognize bacteria in the female reproductive tract, this is an emerging research interest field. Bacterial infections of the female reproductive tract, including vaginitis, cervicitis, and endometritis, have been described [159], as this pathogenic environment may cause inflammation and immune activation in the endometrium, impairing embryo implantation and the onset of a successful pregnancy [159,160].

The interplay between nutrition and infectious diseases has been identified. In the era before antibiotics, the diet was a vital part of controlling infections [161]. Malnutrition, including undernutrition and overnutrition, can increase sensitivity to infectious diseases and magnify the infection severity, which can worsen by malnutrition; the gut microbiota has been attracting interest as an essential mediator in the complex relationships linking food, the human body, and infectious diseases [162].

An optimal vaginal microbiota is controlled by Lactobacillus species, which produce the metabolite lactic acid. Lactic acid decreases the pH of the vaginal microenvironment [163] and, throughout immunomodulatory and direct inhibitory effects, may defend against the acquisition of STIs, including Chlamydia trachomatis (CT) and HIV [164,165]. Women with a non-optimal microbiota, as epitomized by the clinical condition of bacterial vaginosis (BV), have vaginal microbial communities low in Lactobacillus spp. and are instead colonized by a variety of anaerobes that generally produce little or no lactic acid. Some of these bacteria produce metabolites such as biogenic amines and short-chain fatty acids that may be pro-inflammatory and linked with symptoms such as vaginal malodor and discomfort. These metabolites may also increase susceptibility to STIs. Moreover, women with a low-Lactobacillus non-optimal vaginal microbiota have an increased risk of being infected with STIs and ascending infection, including pelvic inflammatory disease (PID) and increased risk of preterm birth (PTB) [166].

Bacterial vaginosis (BV) is the most common reason for vaginal complaints amongst reproductive-age women. The prevalence of BV in infertile women is high (19%), and an abnormal microflora occurs in 39% of infertile patients [167,168]. BV is a clinical case marked by a transformation from a Lactobacillus-dominant bacterial community to higher diversity and a greater abundance of anaerobes and a subsequent rise in vaginal pH (>4.5) [169,170].

BV is considered a risk factor for several common sexual transmitted infections [171], including those induced by the bacteria Neisseria gonorrhea, CT, and Mycoplasma genitalium; the protozoan Trichomonas vaginalis; and viruses such as HIV, human papillomavirus (HPV), and herpes simplex virus type 2 (HSV-2) [158,169,170]. Many data have reported the relationship between diet and nutritional status in BV, but the mechanism is still unclear [162,172]. Many studies have found associations between BV and low micronutrient status, including vitamins A, C, E, and D and β-carotene, and low dietary intake of folate and calcium [162,170,172,173,174].

### 6.1. Bacterial Vaginosis and Vitamin D Deficiency

Many records describe higher frequencies of BV among women with low vitamin D concentrations (often marked as <20 nmol/L or <30 nmol/L) [175,176]. In addition, vitamin D supplementation is effective in eliminating BV [177]. Race/ethnicity has significant population-level impacts on vitamin D status, BV status, and pregnancy outcomes. Women of African heritage are also doubly as likely to receive a clinical diagnosis of BV, and analyses of the vaginal microbiota reveal that it is more likely to be colonized by specific BV-associated bacteria.

### 6.2. Role of a High-Fat Diet and a High-Sugar-Diet on Altering the Vaginal Microbiome

In specific subsets of women, a correlation between a high-saturated-fat diet, a higher glycemic load, and lower nutritional density with BV has been found, in addition to a contrary relationship between BV and higher folate, vitamin E, and calcium consumption [170,172,178]. BV has also been epidemiologically combined with obesity [169]. Subsequently, shifts in the vaginal microbiota balance due to infection with BV alters the composition referred to as polybacterial dysbiosis and to disease such as vaginal HPV [179]. In addition, BV has been associated with acquiring and transmitting HIV and other sexually transmitted pathogens [180,181].

**Figure 1:**
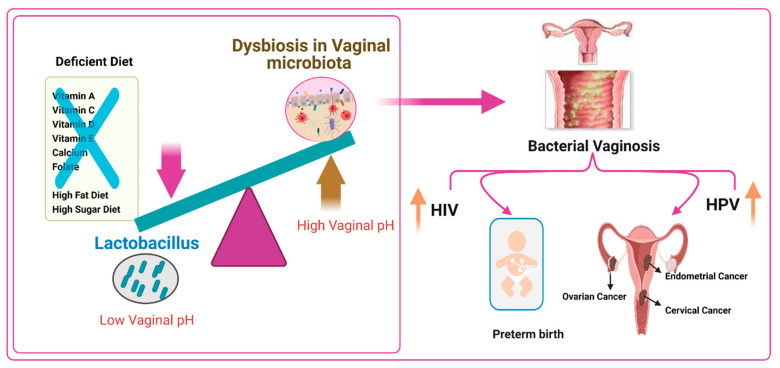
Impact of diet on the vaginal microbiome. A diet deficient in vitamins A, C, D, and E, calcium, folate, and beta-carotene but loaded with fats and sugar leads to altering the vaginal microbiota and increase susceptibility to infections causing bacterial vaginosis, which are associated with preterm birth, raising the risk of human immunodeficiency virus (HIV) transmission, human papillomavirus (HPV) infection, and cervical, endometrial, and ovarian cancers.

Lactobacillus dominance is that the high starch content of human diets leads to high glycogen levels in the vaginal tract, creating a suitable Lactobacillus environment. Lactobacilli and other fermentative bacteria and vaginal epithelial cells produce lactic acid and are responsible for acidifying the vaginal microenvironment pH to <4.5, which gives the vaginal microbiota a certain level of balance and ability to withstand some infections. This microbiota is shown by a low degree of diversity and the high dynamics of its structure changes under the control of various exogenous and endogenous factors. Nutrients play an important role in altering the vaginal microbiome diversity. A diet deficient in vitamin A, C, D, and E, calcium, folate, and beta-carotene but rich in fats and sugar, causes vaginal infections such as BV, which are linked to preterm birth, increased risk of HIV transmission, increased risk of HPV infection, and cervical, endometrial, and ovarian cancers (Figure 1).

## 7. Gynecological Cancers

Neoplastic diseases are a growing public health problem, considering their incidence and subsequent health care burden [182,183]. Interdisciplinary oncological care is extremely important for the sake of successful cancer cure. Adequate prophylaxis, diagnostic work-up, and therapeutic plan are essential in order to either achieve a curative goal in the case of curable diseases or prolong patient survival and quality of life in the case of an incurable disease. In gynecological oncology, the utility of prophylaxis and health-promoting behavior strategies can result in three outcomes: effective, leading to poor effects, or ineffective. Examples of the first outcome are vaccination and the application of effective screening tools in cervical cancer, with subsequent reduced morbidity and mortality [184]. Intermediate effective outcomes are achieved in endometrial cancer, where behaviors such as avoiding aggravating factors and following their effective treatment in co-morbid conditions (diabetes, hypertension, and obesity) reduced morbidity in some groups [185]. Unfortunately, following the same measures in the case of ovarian cancer resulted in practically ineffective outcomes [186].

Diet and nutritional intervention plans in oncology should be individualized and focused on adjusting nutrient needs for cancer patients [187]. The literature estimates that diet and nutritional compounds may contribute to approximately 20–60% of cancers worldwide [188]. Collectively, more investment should be made in research detailing the role diet/nutrition plays both in the occurrence of cancer and in its cure, such as tolerance to radiation and chemotherapy. The current literature relatively ignores this important angle, while focusing on exploring new chemotherapeutics, immunological therapies, and new operative techniques, including those only for women [189]. Recent findings have shown that neither fruits nor vegetables might be convincingly or possibly associated with the risk of any cancer. Moreover, vitamins and mineral compounds do not reduce cancer risk in populations that are well nourished. Therefore, they should not be used for standard cancer prevention. However, specific components of certain fruits and vegetables might present protective properties [189]. Seemingly, even if studies in this area are scarce, it is worth collecting at least some available data to draw conclusions for future research.

### 7.1. Cervical Cancer

Cervical cancer has been studied for many years. Unlike other cancers, cervical cancer is caused by sexually transmitted infections (STIs) with certain types of HPV. A persistent viral infection in high-risk groups was recognized as necessary for the development, maintenance, and progression of cervical intraepithelial neoplasia (CIN) and cervical cancer [190]. Some environmental and lifestyle co-factors were found to influence such disease progression, including inappropriate diet, cigarette smoking, coexistence of STIs, combined oral contraceptive (COC) use, high parity, low socioeconomic status, early sexual activity, or multiple sexual partners.

Available studies have demonstrated that reactive oxygen species (ROS), either independently or orchestrated with HPV, may play a role in cervical cancer pathogenesis. Therefore, consumption of dietary antioxidants, such as carotenes, ascorbic acid, and vitamin D, might offer a protective role via neutralizing such harmful ROS [188]. Moreover, antioxidants may modulate the immune system in favor of a better response to the cancer microenvironment [191].

Likewise, natural antioxidants may slow down or protect against persistent HPV infection and thus later cervical cancer development [192,193]. For example, Tomita et al. highlighted an existing correlation between both low intake of fruits and vegetables and smoking habits with an increasing risk of developing high-grade CIN [192]. In addition, in 2012, Jia et al. published data suggesting that eating higher amounts of fresh vegetables and drinking green tea may reduce the overall risk of cervical cancer [194]. Interestingly, vitamins such as C and E can serve as efficient antioxidants, with studies linking higher serum antioxidant vitamin levels with a lower risk of cervical cancer, especially in passive smokers [195]. It was also demonstrated that the dietary intake of carotenoids or retinols, lutein, different xanthines, and ascorbic acid was associated with a reduced incidence of HPV persistence in infected women [196]. Furthermore, low serum levels of lycopene, retinol, and tocopherols are suspected to increase the risk of high-grade CIN, while higher serum levels of carotenoids and gamma tocopherol could even reduce the overall risk of this kind of dysplasia [192].

A summarized review by Ono et al. in 2020 concluded that various nutritional antioxidants may affect HPV infection-derived cervical diseases. They suggested that the intake of vitamin A, carotenoids, and vitamin D may inhibit cervical cancer development at early stages. Conversely, the intake of vitamin C and E may be useful in the inhibition of the cervical cancer development process [197], while vitamin A’s main effect is inhibition of HPV infection and CIN development [188]. In 2000, Yeo. et al. found that women with low serum retinol levels were at an increased risk of low-degree CIN compared with women who had higher levels of retinol [198]. Interestingly, Huang et al. (2020) found that the dietary intake of vitamin A, equal to or higher than 1448.155 mcg, increased the risk of HPV infection by 70% [199]. Vitamin E is a group of fat-soluble compounds including tocopherols and tocotrienols with antioxidant effects protecting cell membranes from ROS [200]. Studies have confirmed the same findings that high circulating levels or a greater intake of vitamin E might reduce the risk of CIN or cervical cancer [201]. Concerning other nutrients such as vitamin D and folic acid, a recent randomized controlled trial by Vahedpoor et al. (2017) performed in 58 women diagnosed with low-grade CIN revealed that after six months of vitamin D administration, the regression of lesions was observed more commonly in women who consumed vitamin D compared with controls [202]. Folic acid has not been extensively studied recently, and the available data are relatively old. Hernandez et al. (2003) reported that the total folate serum level presented an inverse, dose-responsive correlation with low-grade cervical squamous intraepithelial lesions and high-grade squamous intraepithelial lesions [203].

Natural compounds with chemopreventive/chemotherapeutic potential and antioxidant features have received increased attention in the past few years. Some natural compounds extracted from plants, such as curcumin and EGCG, have been found to exhibit anticancer properties, e.g., increasing tumor cell sensitization to different forms of therapy [204,205]. Seemingly, curcumin requires special attention. This compound (also known as diferuloylmethane) is present in the turmeric rhizome and shows different anti-inflammatory and antioxidant properties [205]. Briefly, curcumin contributes to the inhibition of nuclear factor kappa B-regulated gene factors that control apoptosis, proliferation, invasion, or angiogenesis, in addition to inhibition of nuclear factor kappa B activation through the modulation of different kinases [205], which contributes to the resistance of human cervical cancer cells and results in increased cell death [206]. EGCG is another interesting compound with anticancer properties. It is a polyphenol with proven antiproliferative, antiangiogenic, antimetastatic, and proapoptotic properties in several tumor models [207]. EGCG is a potent inhibitor of several kinases as well as the mammalian target of rapamycin (mTOR) signaling, besides acting as a modulator in inflammatory processes [208]. EGCG modulates ROS production, which might be linked to its antitumorigenic effects. The EGCG-derived modulation/inhibition of nuclear factor kappa B signaling is responsible for its effect against angiogenesis, cell movement, and viability [208]. Finally, resveratrol, which is a phytoalexin found in fruits like grapes, blueberries, or peanuts, exhibits anticancer effects via interacting with several important molecules involved in tumor development, such as activators, kinases, or nuclear factor kappa B. Moreover, resveratrol shows antiproliferative effects on cervical cancer cell lines through cell cycle modulation with accumulation of the cells in the S phase [204].

To conclude, several nutrients with antioxidant effects may present potent abilities to intervene in the natural history of cervical cancer tumorigenesis connected with HPV infection [197]. Selected vitamins (such as vitamins A and D) and natural compounds (e.g., EGCG) demonstrate a positive effect in halting the cervical cancer disease process. Obviously, the available data are inconclusive, and their quality may be undermined. Therefore, more well-designed, large randomized clinical studies are needed.

### 7.2. Endometrial Cancer

Endometrial cancer (EC) is currently one of the most common malignant neoplasms affecting women worldwide. Unfortunately, the direct underlying etiology has not been clearly described and understood. EC is known to occur mainly in postmenopausal women [209]. Contributing risk factors are older age, nulliparity, diabetes, estrogen-only hormone replacement therapy, and obesity [210]. Fortunately, the survival rate ranges from about 75% to 90% in patients diagnosed at early stages [211]. Differences in histological patterns and clinical outcomes divide EC into two types. Type I cancers present as endometrioid adenocarcinomas, and this kind of tumor is often preceded by endometrial hyperplasia. Importantly, the development of this kind of tumor is mostly influenced by the long-lasting stimulation of estrogens on the endometrium [210,212]. Type II tumors are mostly represented by serous cancers, and they are rather estrogen independent. We may observe them arising from the atrophic endometrium [210,212].

Regarding the potential preventive effects of diet intake on EC risk, limited data are available with conclusions of negligible correlation. In their review, Bandera et al. (2009) found an inverse relationship of EC risk with the dietary intake of carotenes, ascorbic acid, and tocopherols. They highlighted that additional larger studies are necessary to confirm this association [213]. Later, a Nurses’ Health Study denied such association between EC risk and the consumption of dietary carotenoids and vitamins A, C, or E [214].

The incidence of precancerous changes was found to be higher in overweight and obese patients. Interestingly, higher fat energy intake was found to be associated with increased EC risk, but the energy from carbohydrates and proteins did not increase that risk [215]. Therefore, it was interesting for researchers to explore the role of diet in the pathophysiology of EC in the context of inflammation. Studies have suggested that elevated levels of prostaglandins might underlie the transformation of a normal endometrium into neoplastic tissue, which might be attributed to inflammation-induced cell division with subsequent possibility of ineffective DNA repair and mutations. Interestingly, polyunsaturated fatty acids (e.g., available in seafood) are thought to be anti-inflammatory, and one might think play a possible beneficial role against EC. For example, Brasky et al. (2014) showed that high consumption of dietary eicosapentaenoic acid and docosahexaenoic acid increased the risk of EC by 80% compared to a lower consumption rate [216]. Alcohol was thought to increase EC risk via increasing the circulating serum sex steroids. Rinaldi et al. (2006) showed that sex-hormone-binding globulin levels are approximately 15% lower in alcohol consumers (25 g of alcohol daily) compared with non-consumers [217]. Interestingly, recent data from a prospective study performed in 301,051 European women showed that alcohol consumption is not associated with EC risk [218].

The effect of some plant-derived compounds on EC is attributed to their hormonal effect, for example, phytoestrogens, which have low estrogenic activity, while others might possess antioxidant and antimutagenic properties [219]. Flavonoids are a class of polyphenolic metabolites that have antioxidant and anti-mutagenic properties, so it is believed that they may reduce cancer risk, e.g., in EC [220]. Unfortunately, it has not been confirmed in further studies. A randomized controlled trial by Wang et al. (2009) revealed no association between selected flavones and flavonols and EC risk [221]. Similarly, Bandera et al. (2009) showed no association between isoflavones and EC [222]. However, slightly different data were presented by Ollberding et al. (2012), who demonstrated that a greater consumption of isoflavone-containing foods was associated with a reduced risk of EC in postmenopausal women [223]. This might be explained by the fact that isoflavones possess some selective estrogen receptor modulator activity, with varying estrogenic and antiestrogenic potential, depending on the receptor characteristics of the target tissue. Isoflavones are abundant in soy, whose consumption has been studied in numerous publications. A review by Zhang et al. (2015) revealed that soy intake might be associated with lower EC risk; however, the authors highlighted that the exact mechanisms are still unknown [224]. Furthermore, a meta-analysis by Zhong et al. (2018) revealed that the consumption of larger amounts of dietary isoflavones from soy products and legumes weakly decreased the risk of EC in the selected population [225]. Remarkably, some authors indicated a negative effect of high soy isoflavone consumption that resulted in a relatively high incidence of endometrial hyperplasia [226]. Therefore, the protective effect of soy isoflavones on EC should be interpreted with caution, and their introduction into cancer therapy might be rather challenging now.

If it is about other compounds, the data are scarce. An inverse association between ultraviolet irradiance and EC incidence was demonstrated, suggesting a possible beneficial effect of vitamin D, considering its multitargeted effects [227]. However, available meta-analyses have revealed no significant relationship between the intake of vitamin D and the incidence of EC [228]. In a study by Bandera et al. (2009), increased intake of quercetin (a bitter plant flavonol found in different fruits and vegetables) was associated with a decreased risk of EC [222]. Similarly, kaempferol, a natural dietary flavonoid, was explored against EC cells, considering its anticancer, anti-inflammatory, and antioxidant properties, and studies have shown that it suppresses cellular proliferation through various mechanisms [229,230].

Some research was conducted on the association between tea consumption and the risk of EC. In 2015, Yang et al. demonstrated little or no association between tea consumption and the risk of EC [231], while a meta-analysis published later highlighted that a higher dietary intake of green tea might be connected with a reduced risk of EC. Notably, such correlation was not demonstrated in the case of black tea. The authors suggested that the reduced risk might be due to the markedly higher content of catechins in green tea in comparison with black tea. Catechins, such as EGCG, may modulate the estrogen-induced activation of endometrial cells and also induce the apoptosis of neoplastic cells as well as cell cycle arrest [232].

Taking all together, available data on the effect of nutritional compounds on EC pathophysiology are of poor quality and insufficient. Nevertheless, exploring new natural dietary compounds in the prophylaxis or treatment of this disease is encouraged.

### 7.3. Ovarian Cancer

Ovarian cancer is a malignant tumor of the ovaries, occurring mainly in peri- or postmenopausal women. Unfortunately, it is associated with the poorest prognosis and the highest mortality rate among all gynecological cancers [233]. Research shows that the number and frequency of ovulations in a woman’s lifetime are linked to the risk of her developing ovarian cancer [234], since it is associated with the rupture of the ovarian epithelium and the sensitizing effect of the follicular fluid with a high content of estrogens [235]. Ovarian cancers are histologically and clinically divided into two different types. Type I cancers are low-grade endometrioid, mucinous, and clear-cell cancers, whereas type II cancers are of a higher histological grade in which tumors may develop de novo from the tubal and/or ovarian surface epithelium and include serous cancers [234]. Surgery is the critical modality in the treatment of ovarian cancer, as well as chemotherapy [234]. Regrettably, due to the unclear etiology of ovarian cancer, it may not always be prevented. However, some factors have been shown to limit the risk of its development, e.g., lactation or the use of combined oral contraception. Therefore, exploring the potential role of nutritional compounds in prophylaxis or supportive treatment is valid.

The available literature suggests a possible link between ovarian cancer and inappropriate dietary habits. For instance, chronic inflammation was implied as an underlying mechanism contributing to ovarian carcinogenesis [236]. A study by Shivappa et al. (2016) showed that ovarian cancer risk increases in women who consume higher amounts of pro-inflammatory products [237]. On the contrary, Dolecek et al. (2010) and Playdon et al. (2017) demonstrated the influence of a healthy diet on the clinical course of ovarian cancer [238,239]. The former study showed that only yellow and cruciferous vegetables significantly favored the survival rate, whereas a negative correlation was shown for meat [238]. Playdon et al. (2017) demonstrated a trend toward lower mortality with higher fruit intake. Moreover, a higher intake of green leafy vegetables was inversely associated with mortality. Compared to the previously discussed study, the authors did not show such a strong influence of cruciferous vegetables [239]. An important meta-analysis published by Qiu et al. revealed that a high consumption of total, saturated, and trans fats increased ovarian cancer risk. The authors emphasized that different histological subtypes had different susceptibilities to dietary fat, and provided an example of saturated fats that might increase the overall risk of serous and endometrioid ovarian cancers [240].

A variety of studies are available considering phytoestrogens, including the beneficial effect of isoflavones on ovarian cancer, with nonconclusive findings. In a study by Bandera et al. (2011), phytoestrogen presented a trend for a reduction in ovarian cancer risk. However, no significant associations were found. However, an inverse association with total phytoestrogen consumption was found after adjusting for age, race, education, body mass index, and total energy [241]. Moreover, an analysis by Neill et al. (2014) showed a pattern of inverse associations between the increasing intake of phytoestrogens, isoflavones, or lignans and the risk of ovarian cancer, but it should be emphasized that significance was only proved for two lignans—matairesinol and lariciresinol [242]. Isoflavones were found to have a protective effect against ovarian cancer, which may be attributed to the inhibition of the growth and proliferation of ovarian cell lines. Furthermore, they may regulate cancer inflammation pathways [188]. Conversely, Hedelin et al. (2010) found no association between phytoestrogens, fiber intake, and ovarian cancer risk. The authors found that fiber and coumestrol intake was inversely associated with borderline tumors but not with invasive types [243]. Finally, in 2016, Hua et al. showed in their meta-analysis that the intake of dietary flavonoids might decrease ovarian cancer risk. According to this analysis, ovarian cancer risk decreases with isoflavones and flavonols, but there was no evidence that the dietary intake of flavones was protective in the case of ovarian cancer [244].

Herein, we present some examples of such flavonoids that might offer beneficial effects against ovarian cancer. Quercetin, a plant flavonol, inhibits oxidation and acts as a free-radical scavenger with estrogenic activities on both types of estrogen receptors (α and β) [87]. Quercetin presented antitumor and anti-inflammatory properties with a cytotoxic influence on ovarian cancer, which Shafabakhsh et al. attributed to its anti-inflammatory, pro-oxidative, antiproliferation, and apoptosis induction activities [245]. A different already described flavonoid, kaempferol, was also found to be a good inhibitor of angiogenesis [246]. Finally, a flavonol named galangin was found to be selective against cancer cells where it induced apoptosis. It was suggested that future research might prove its usability in platinum-resistant ovarian cancers [247].

Curcumin is a well-known natural compound found in turmeric. It is a diarylheptanoid and belongs to the group of curcuminoids, which are natural phenols. Curcumin exhibited a wide range of effects, e.g., anticancer, anti-inflammatory, and antioxidant capabilities. In 2007, Lin et al. showed that curcumin might inhibit nuclear factor kappa B activation and suppress both proliferation and angiogenesis in ovarian cancer cells [248]. Since curcumin has been extensively studied in cancer treatment, data might suggest additional efficacy due to sensitization of the resistance of cancer cells to current therapies [249]. For example, a study conducted by Wahl et al. (2007) demonstrated that curcumin used in a combination with a special anticancer ligand (Apo2L) results in reduced chemoresistance to conventional chemotherapeutic agents [250]. More recently, He et al. (2016) found that curcumin significantly increases epithelial ovarian cancer sensitivity to cisplatin and abolishes the sphere-forming ability [251]. An earlier study by Yallapu et al. in 2010 showed that this compound reduces the dose of both radiation and cisplatin needed for cell growth suppression in cisplatin-resistant ovarian cancer cells [252]. Berberine is a plant-based alkaloid with a tetracyclic skeleton with anti-inflammatory, antiproliferative, pro-apoptotic, and antimetastatic actions [253]. A recent study by Liu et al. (2019) proposed that the co-use of berberine and cisplatin enhances ovarian cancer cell death by inducing apoptosis and necroptosis. Tissue samples revealed the typical apoptotic and necrotic cell death morphology with the inhibition of proliferating cell nuclear antigen and Ki67 and a higher expression of selected caspases [254].

Finally, we highlight new agents that might be of interest for future research on ovarian cancer therapy. The first is honokiol, a natural biphenolic lignan extracted from different parts of magnolia. Regarding the possible effects on ovarian cancer, honokiol regulates the nuclear factor kappa B activation pathway and VEGF expression [255]. Recently, a study showed that the anticancer activities of honokiol in ovarian cancer cells are mediated through the activation of adenosine 5’ phosphate-activated protein kinase. Honokiol induced apoptosis with the activation of various caspases. Moreover, honokiol inhibited the migration and invasion of ovarian cancer cells [256]. The second new compound is bufalin, which is a steroid isolated from toad venom. According to available studies, bufalin presents antitumor effects against various malignancies, including lung cancer [257]. A study by Su et al. published in 2020 demonstrated its usefulness in ovarian cancer, where it acted as a potent inhibitor of cell growth and migration in ovarian cancer cells through the suppression of mTOR activation and hypoxia-inducible factor 1-alpha (HIF1α) induction. The authors concluded that bufalin might be used as an additive to cisplatin in ovarian cancer therapy [258]. Lastly, tetramethylpyrazine (also named ligustrazine) is a chemical compound classified as an alkylpyrazine found in fermented soybeans and cocoa beans [259]. It is a natural compound reported to present anticancer activity. In 2020, Zhang et al. found that tetramethylpyrazine inhibits the viability, proliferation, migration, and invasion ability of selected ovarian cancer cell lines in a dose-dependent manner [260].

Vitamin D may be significant in reproductive organ tumors [261]. A systematic review of the literature has not identified any human studies regarding the effect of vitamin D or its analogues on ovarian cancer patients, and such supplementation or treatment cannot be recommended [262]. Regardless of vitamin D, calcium seems to be significant in the pathophysiology and therapy of ovarian cancer. An available meta-analysis by Song et al. published in 2017 supported the hypothesis that increased calcium intake might reduce ovarian cancer risk. In the analysis, dietary calcium was significantly associated with a reduced risk of ovarian cancer among cohort and case–control studies. However, the authors concluded that further studies, mostly those on larger groups, might lead to more decisive conclusions [263].

Although the discussed data indicated some influence of nutritional compounds on the development and course of ovarian cancer, there is a paucity of valuable clinical data that may be translated into evidence-based medicine. Flavonoids seem to play the most significant role. However, more research is encouraged in order to explore novel compounds.

## 8. Menstrual Disorders

### 8.1. Menorrhagia

Menorrhagia is described as excessive uterine bleeding, in terms of flow and duration, during regular cyclical intervals. Its clinical definition includes blood loss greater than 80 mL per cycle or menses lasting longer than 7 days [264]. Diet should be considered when managing menorrhagia. Ideally, the diet should be low in animal fat and rich in fish oils and linolenic and linoleic acids. Therefore, flaxseeds and soy protein have been frequently suggested due to their ability to regulate the menstrual cycle [265]. Here, we briefly discuss supplements and nutrients that have been explored for their potential role in managing menorrhagia.

#### 8.1.1. Iron

Blood loss is one of the major causes of iron deficiency anemia. However, it is less well known that chronic iron deficiency can be a contributor to menorrhagia, in turn. Therefore, women experiencing heavy blood loss should consume iron-rich foods such as Brewer’s yeast, wheat germ, blackstrap molasses, organic liver and kidneys, apricots, eggs, ground beef, raisins, beans, cooked spinach, and chicken. In addition, yogurt, sour fruits, and citrus juices can aid in the absorption of iron [266].

#### 8.1.2. Vitamin A

Adult women experiencing menorrhagia may have low levels of vitamin A. One study in which vitamin A was used to treat women with menorrhagia showed that those who received 60,000 IU of vitamin A for 35 days experienced both a return to normal and a reduction in blood loss compared to the placebo group [264,267].

#### 8.1.3. Vitamin B Complex

Vitamin B deficiency may be related to menorrhagia. Studies have shown that vitamin B complex deficiencies result in failure of the liver to inactivate estrogen. Thus, the excess estrogen’s effect on the endometrium ends up with more bleeding, while vitamin B complex may help normalize estrogen metabolism and thus reduce bleeding [268].

#### 8.1.4. Vitamin C and Bioflavonoids

Vitamin C and bioflavonoids improve heavy bleeding via making the capillary walls less fragile. Livdans-Forret noted that 16 of 18 women who took vitamin C and bioflavonoids for heavy menstrual bleeding reported an improvement [264]. Moreover, vitamin C can benefit women with iron deficiency due to menorrhagia by increasing iron absorbency [269].

Some herbal and nutritional supplements have shown beneficial effects against menorrhagia, including the chaste tree or chasteberry (Vitex agnus castus), which is a well-known herb in Europe for the treatment of hormonal imbalances and abnormal bleeding in women. In addition, astringent herbs such as shepherd’s purse have a long history of use for inhibiting gynecological hemorrhage. Tonic herbs such as life root, also known as ragwort, have been used for conditions such as menstrual cramps, menorrhagia, and subdued menstruation. Traditional herbs such as yarrow have been used since medieval times to treat bleeding wounds. Yarrow is a uterine stimulant that increases muscular tone, stimulates reproductive activity, and effectively treats menstrual problems [264].

### 8.2. Dysmenorrhea

Dysmenorrhea is commonly described as painful menstruation in the form of lower abdominal pain, which has a range of severity and associated symptoms. These include nausea, vomiting and loss of appetite, fatigue, diarrhea, headache, restlessness, insomnia, and fainting [270]. Primary dysmenorrhea has been primarily associated with the extra production of prostaglandins and leukotrienes. Prostaglandins (PGF2-α) temporarily limit or stop the blood supply to the uterus by stimulating its contraction, which reduces the amount of blood perfusing the uterus through myometrial compression of the blood vessels. This deprives the uterus of oxygen, which results in cramping and abdominal pain. Higher concentrations of PGF2-α and leukotrienes in menstrual blood and in uterine smears were observed in women with signs of painful menstruation. Several studies have explored the efficacy of supplements and nutrients against dysmenorrhea [271,272,273], which we discuss next in the article.

#### 8.2.1. Calcium and Magnesium

Dietary calcium and magnesium intake has a protective effect against dysmenorrhea. Following absorption from the upper intestine, they can manage the muscle cells’ response to nerve stimuli through numerous functions [274]. Even though the tocolytic effect of magnesium has already been proven in vivo and in vitro, the best dosage for treating or preventing dysmenorrhea is not yet clear [275].

#### 8.2.2. Olive Oil

The polyphenolic compound oleocanthal in extra virgin olive oil has been shown to have anti-inflammatory and antioxidant effects. A study examining its inhibitory effect on prostaglandin-induced uterine hypercontraction showed that oleocanthal, dose-dependently, inhibited the PGF2α-induced contraction amplitude [276]. Thus, the authors concluded that extra virgin olive oil and oleocanthal can reduce oxidative stress and uterine hypercontraction.

#### 8.2.3. Fennel

Fennel, or Foeniculum vulgare, is a herbal therapy proposed to alleviate menstrual pain by lowering the prostaglandin levels in blood. A meta-analysis showed the equivalent effects of fennel on pain reduction compared with drug therapy, and the pooled results showed favorable effects of fennel on pain reduction compared to the placebo [277]. Fennel in the form of capsules, pill, or oils (excluding massage oil) was used in the 12 studies included in the meta-analysis.

#### 8.2.4. Dietary Fiber

Since dietary fat and fiber alter estrogen levels, they may be related to dysmenorrhea by affecting hormones. Fiber intake reduces blood estrogen levels, whereas fat has been associated with increased estrogen levels. Nagata et al. found that intake of dietary fiber is significantly inversely correlated with the menstrual pain scale after adjusting for age, smoking status, age at menarche, and total energy intake [278].

#### 8.2.5. Omega-3 and Omega-6 Fatty Acids

Western diets are rich in omega-6 fatty acids (e.g., vegetable oil, eggs, and margarine) but poor in omega-3 fatty acids (e.g., fish, canola oil, and wheat germ). Omega-6 fatty acids contribute to the formation of pro-inflammatory eicosanoids, such as Prostaglandin E2 (PGE2), thromboxane A2, and leukotriene B4, whereas omega-3 fatty acids, specifically eicosapentanoic and docosahexanoic acids, lead to the formation of less inflammatory eicosanoids (e.g., PGE3, thromboxane A3, and leukotriene B5). There is some epidemiologic evidence that a diet rich in omega-3 fatty acids can decrease painful menses. Several studies have shown a significant decrease in menstrual pain in those using fish oil [279,280].

#### 8.2.6. Vitamin D

Vitamin D receptors are located in the human uterus, and vitamin D inhibits the synthesis of prostaglandins [144]. Calcitriol (1,25[OH]2D) decreases, in vitro, the level of pro-inflammatory cytokines such as interleukin 6 and tumor necrosis factor and regulates the expression of several key genes involved in the prostaglandin pathway, causing decreased biological activity of prostaglandins [144]. Thus, vitamin D has been suggested to halt the extra prostaglandin production found in primary dysmenorrhea. One study showed an inverse correlation of 25(OH)D levels with the pain score as well as a significant reduction in pain in women taking vitamin D, with the greatest reduction found in women who reported severe pain at baseline [281]. Studies in Iran and Italy have shown that the use of a single dose of oral cholecalciferol (300,000 IU) for five days before the beginning of menstrual bleeding significantly decreased the pain of severe primary dysmenorrhea, while another trial found that the administration of 50,000 IU of vitamin D for eight weeks significantly reduced pain severity [282]. Another study found that low levels of vitamin D are inversely related to the severity of primary dysmenorrhea and that vitamin D and calcium intake could reduce its severity [283].

#### 8.2.7. Vitamin E

Vitamin E is thought to reduce prostaglandin formation by inhibiting arachidonic acid release. A review article about the positive effects of vitamin E on the alleviation of primary dysmenorrhea pain showed a significant reduction in pain severity in women treated with this vitamin [264]. Two studies have shown a significant reduction in pain when 150 to 500 IU/day of vitamin E was administered a few days before and during menses compared with the placebo for two to three cycles [264].

#### 8.2.8. Qixuehe

Formulations of Chinese herbs may be beneficial but lack rigorous testing to evaluate their mechanistic action. One study found that QiXueHe Capsule (QXHC) can alleviate pathological changes in menstrual disorders. Researchers identified 1022 targets of 15 herbs in QXHC to investigate its pharmacological mechanisms on menstrual disorders. Results showed that targets in the treatment of menstrual disorders are significantly associated with several biological pathways, such as VEGF and chemokine signaling pathways and alanine, aspartate, and glutamate metabolism, which are involved in the major pathological processes of menstrual disorders. The authors also found 20 pairs of QXHC candidate targets, and the corresponding chemical components had the strong binding free energy. These results showed that the pharmacological mechanisms of QXHC in the treatment of menstrual disorders may be associated with its involvement in hemopoiesis, analgesia, nutrient absorption and metabolism, mood regulation, as well as immune modulation [284].

#### 8.2.9. Zinc

Zinc has been found to reduce the synthesis of prostaglandins via its action as an endogenous antioxidant catalyst and as an anti-inflammatory agent that can improve microcirculation of endometrial tissue. This was shown in a study that found that zinc significantly lowered the pain duration and severity in women compared to the control group and improved the patients’ quality of life [285]. One study suggested that daily intake of 30 mg of zinc one to four days prior to menstruation can prevent menstrual cramping pain, without harmful side effects, while another showed evidence that zinc can treat primary dysmenorrhea in adolescent girls [286].

#### 8.2.10. Vitamin K

A few studies have investigated vitamin K (phylloquinone) injection to treat primary dysmenorrhea. Treatment with vitamin K may shorten the length of the extended menstrual flow due to its action on prothrombin, which is a coagulation protein produced in the liver and is dependent on vitamin K [287]. Chao et al. reported that women indicated a significant decrease in pain after vitamin K injection in both legs and increased plasma phylloquinone levels [287]. Wade et al. noted that both women given vitamin K3 using an acupuncture point injection or deep muscle injection experienced a decrease in average pain as well as menstrual distress [288]. It was suggested that women with severe primary dysmenorrhea could manage severe dysmenorrhea with two vitamin K acupuncture point injections per year.

Finally, an interesting recent study examined the relationship of breakfast to the development of future reproductive diseases. Missing this first meal interferes with the start of the active phase during the circadian rhythm that is regulated by the central clock system. Since both food intake and the light/dark cycle are the main regulators of circadian rhythms, skipping breakfast can lead to changes in light stimulation within the central clock system [289]. The authors suggested that meal skipping affects the hypothalamic–pituitary–ovarian axis, impairs the reproductive rhythm, and leads to ovarian dysfunction. Young women who skip breakfast show significantly higher incidences of dysmenorrhea and irregular menstruation, suggesting that missing meals affects ovarian and uterine functions [289]. Since dysmenorrhea becomes more manifested in those with a history of dieting, the authors posited that inadequate dietary habits in adolescence become a trigger for the subsequent development of organic gynecological diseases. [289]. Thus, they suggested shifting the focus from therapeutic to prophylactic and from dietary content to dietary timing in the management of gynecological disorders in young women. Further investigation, together with developing new methods, is recommended to test their hypothesis.

## 9. Conclusions

Gynecological diseases, like other diseases, have a causal relationship with some factors in the environment. These factors may be physical or/and social. Someone may suffer from gynecological diseases either due to her physical condition/exposure (e.g., nutritional status, environment, exposure to bacteria or viruses, etc.) or due to social conditions (education, income level, culture, etc.). So, while dealing with a gynecological disease clinically, it is recommended to look at these factors that might improve the outcome. In this article, we covered several dietary supplements and nutrients that may provide potential benefits upon implementation in preventive/therapeutic measures to control common gynecological diseases, including uterine fibroids, endometriosis, PCOS, infertility, menstrual disorders, and vaginal infections, as well as malignant cancers such as cervical cancer, endometrial cancer, and ovarian cancer. Nutrition has the most important lifelong environmental impact on human health. There are several studies indicating that fruits, tea, vegetables, as well as various dietary compounds can alter several signaling pathways involved in disease pathogenesis as well as impact cancer cells, such as the activation of tumor suppressor genes and an increase in apoptosis and the activity of cell survival proteins, thus playing a protective role against cancer. However, this research area needs more attention.

## Data Availability

Not applicable.
